# Predicting synchronous firing of large neural populations from sequential recordings

**DOI:** 10.1371/journal.pcbi.1008501

**Published:** 2021-01-28

**Authors:** Oleksandr Sorochynskyi, Stéphane Deny, Olivier Marre, Ulisse Ferrari

**Affiliations:** 1 Sorbonne Université, INSERM, CNRS, Institut de la Vision, 17 rue Moreau, F-75012 Paris, France; 2 Current affiliation: Department of Applied Physics, Stanford University, Stanford, California, United States of America; Stiftung caesar, GERMANY

## Abstract

A major goal in neuroscience is to understand how populations of neurons code for stimuli or actions. While the number of neurons that can be recorded simultaneously is increasing at a fast pace, in most cases these recordings cannot access a complete population: some neurons that carry relevant information remain unrecorded. In particular, it is hard to simultaneously record all the neurons of the same type in a given area. Recent progress have made possible to profile each recorded neuron in a given area thanks to genetic and physiological tools, and to pool together recordings from neurons of the same type across different experimental sessions. However, it is unclear how to infer the activity of a full population of neurons of the same type from these sequential recordings. Neural networks exhibit collective behaviour, e.g. noise correlations and synchronous activity, that are not directly captured by a conditionally-independent model that would just put together the spike trains from sequential recordings. Here we show that we can infer the activity of a full population of retina ganglion cells from sequential recordings, using a novel method based on copula distributions and maximum entropy modeling. From just the spiking response of each ganglion cell to a repeated stimulus, and a few pairwise recordings, we could predict the noise correlations using copulas, and then the full activity of a large population of ganglion cells of the same type using maximum entropy modeling. Remarkably, we could generalize to predict the population responses to different stimuli with similar light conditions and even to different experiments. We could therefore use our method to construct a very large population merging cells’ responses from different experiments. We predicted that synchronous activity in ganglion cell populations saturates only for patches larger than 1.5mm in radius, beyond what is today experimentally accessible.

## Author summary

A major goal of neuroscience is to understand how entire populations of neurons process sensory stimuli. This understanding is limited because current experimental techniques do not allow to record all relevant neurons of a sensory structure. A possible strategy to overcome this issue, is to use sequential recordings from cells recorded in multiple experiments to reconstruct how the entire population will respond. Yet this approach can not account for collective behaviour and synchronous activity within the population. Here we address this issue in the retina and propose a method to infer the activity of an entire population of neurons of the same type from sequential recordings. Our method assumes that we have access to many single cell recordings gathered from different experiments, and additionally to a few synchronous recordings of pairs of neurons of the same type. By combining copula distributions and maximum entropy modeling, we used these data to reconstruct the collective activity of a large population of neurons and to show how synchrony grows with the number of neurons.

## Introduction

A major goal of neuroscience is to understand how populations of neurons process sensory stimuli. This understanding is limited because, among other reasons, accessing the activity of all neurons of a sensory structure is very challenging. Most techniques only give access to a small fraction of neurons [1, 2] (but see [3, 4]), leaving as hidden variables many neurons that may play a role in information processing but are not recorded.

To overcome this issue, an emerging, ‘divide and conquer’ approach is to first classify the neurons in a given area into different cell types, where neurons of the same type are supposed to be functionally identical. Then, in a second step, one can characterize the neuronal function of each cell type, to eventually predict how populations composed of all the neurons of the same type will respond to sensory stimuli.

There has been tremendous progress recently in achieving the first step of this approach. Several studies have shown that it is possible to cluster cells in different homogeneous types [5]. This can be done using either the responses of each cell to several standard stimuli [6–8], or using genetic tools [9, 10]. These methods have proven successful in isolating most cell types in the retina [7, 11, 12] and there are several ongoing studies trying to apply these approaches in the cortex [13].

For the second step, many studies have tried to model and predict how neurons of a single type respond to complex stimuli. This strategy has been applied in the retina [14–18] and in many low-level areas [19–21]. A complementary strategy is to use mouse lines expressing GFP in specific cell types in order to record sequentially (i.e. repeatedly across different experiments) from cells that are functionally identical. This has been performed in the retina [6, 22] and enables one to present as many stimuli as desired to cells belonging to the same type. It is thus possible to gather a lot of information about how single neurons of a well-defined cell type will respond to many different sensory stimuli, using sequential recordings of neurons of the same type taken from different experiments.

Extensive characterization of single cell responses to sensory stimuli is thus possible. The next challenge is to infer how the entire ensemble of neurons of a single type responds together to stimuli. Ideally, one would like to record from all the neurons of a given type, but this is rarely possible.

One possible strategy is to use these sequential recordings from cells of the same type to reconstruct how the entire population will respond. However, reconstructing the activity of a full population from sequential recordings cannot be done by simply pooling the responses to a given stimulus from many sequential recordings. In many cases, pairs of neurons are correlated due to shared noise (noise correlation), which might significantly reshape the neurons’ activity [23], and play an important role in information encoding and transmission [15, 24–29]. Because these noise correlations cannot be predicted from sequential recordings, a model is needed to predict them and therefore to infer the activity of a full population of neurons of the same type.

Here we address this issue and propose a method to infer the activity of an entire population of neurons of the same type from sequential recordings in the retina. Our method assumes that we have access to many single cell recordings gathered from different experiments of neurons of the same type, where the same stimulus has been displayed, and additionally to a few recordings of pairs of neurons of the same type. We used these data to reconstruct the activity of a large population of neurons, with a shared noise consistent with the paired recordings.

Previous works have built models capable of predicting the activity of ensembles of neurons in the retina [15, 30–35]. However, they were fitted and tested on an ensemble of neurons recorded simultaneously, and it is unclear if they can generalize to predict correlated activity across experiments, a critical feature necessary to reconstruct activity from sequential recordings. Other approaches [36–40] have focused on predicting missing correlations in a dataset of multiple neuronal ensembles. However, most of these works rely on some overlap between two simultaneously recorded populations to predict correlations for neurons that are not recorded together, while our method is capable of predicting correlations across experiments.

We applied this method in the rat retina, where the activity of many neurons of the same type can be recorded through repetitions of multi-electrode array experiments [3], so that the method can be validated. We first show that a copula-based analysis [41–45] of synchronous activity allows a simple description of noise correlations, that is invariant across stimulus identities and experimental preparations. This description depends only on the individual activities of pairs of cells and on their physical distance. This result allowed us to construct a model based on copulas and to predict -across experiments- the extent of pairwise noise correlations from sequential recordings of neurons of the same type. From this estimation of pairwise correlations we then used a time-dependent maximum entropy model [33–35] to infer the activity of the full population of neurons of the same type. We show that this method is accurate and reproduces several features of the recorded population activity. We then applied our method to infer the activity of a large population of neurons of the same type, beyond what can be currently recorded experimentally. Thanks to our inference method, we could estimate the extent of synchronous firing in such a large population, and show that it first grows significantly to then saturate for large populations of about 200 neurons.

## Results

### Overview of the inference method

The purpose of our method is to reconstruct the activity of a population of neurons of the same type from their individual responses to a same stimulus. Part of this population activity is directly accessible from sequential recordings, but another part needs to be predicted. For example, if we have recorded sequentially two neurons responding to the same stimulus, a naive solution is to pool together their responses as if they had been recorded at the same time. If the noise present in these responses is independent between the two neurons, this is indeed equivalent to recording them together. However, in many cases, the noise between different neurons is correlated. In that case, pooled sequential recordings are not equivalent to simultaneous recordings [23], and the difference is what is usually termed the noise correlation between these two neurons.

Our method aims at inferring these noise correlations from parsimonious pairwise recordings of a few cells, and use them to predict how noise will be correlated across an entire population of hundreds of neurons. From this we can reconstruct how a large population of neurons would respond if they were recorded simultaneously, based on sequential recordings.

Our method is divided in three steps. First, we infer the parameter of a model based on copulas [41–43] from simultaneous recordings of few cells. Second, we used the inferred model to predict noise correlations between pairs of neurons from sequential recordings, using only information on the distance between the recorded cells. Our method allows predicting noise correlations for the same pair of neurons responding to a different type of stimulus, and can generalize to predict noise correlations for another pair of neurons of the same type recorded in a different experiment. Third, we use a time-dependent maximum entropy model [33–35] to generalize from pairs of neurons to a full population. This step does not require any additional empirical information with respect to the second step. Note that simultaneous recordings are necessary only for model inference (and validation), yet sequential recording are sufficient for making prediction.

Here we applied this method to cells of the same type in the rat retina. These data allowed us to test if our reconstruction of the population activity is accurate. Finally, we used our method to infer the synchronous activity of a large neuronal population, much larger than what is nowadays experimentally accessible.

### Strong noise correlations between nearby OFF retinal ganglion cells

**Fig 1:**
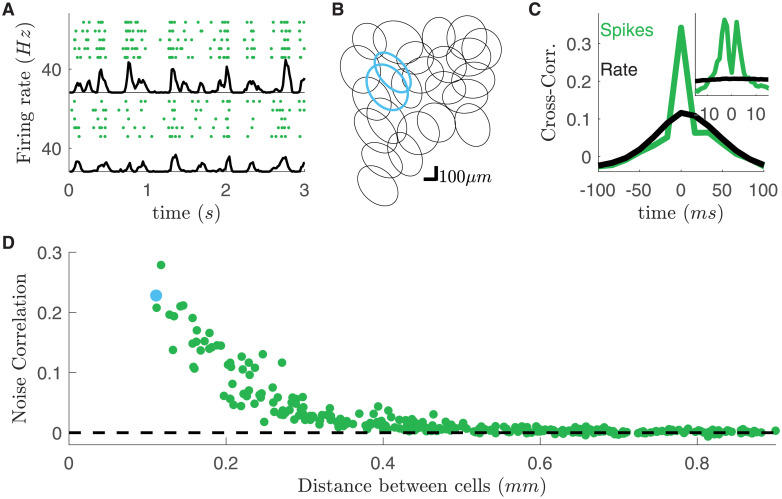
High noise correlation between nearby RGCs subject to checkerboard stimulation. **A**) Raster plots of two example cells in response to checkerboard stimulation. Each line corresponds to a repetition of the same visual stimulation. Black line: averaged firing rate of the cell. **B**) Receptive field mosaic of the recorded OFF cell population. Cyan receptive fields refer to the cells showed in panel A. **C**) Cross-correlation for the two cyan cells (green, *dt* = 17*ms*), superimposed to the cross-correlation of their firing rates (black). Inset: Cross-correlation at finer time scales (*dt* = 1*ms*). **D**) *Zero-lag* (*dt* = 17*ms*) noise-correlation plotted against the distant between cells.

We recorded rat retinal ganglion cells (RGCs) in response to different visual stimuli. We used a previously described method [18] to divide them in different types. Briefly, we clustered their responses to a full field flicker and isolated a single type of OFF-alpha ganglion cells. All these cells responded reliably to a checkerboard stimulus (Fig 1A). The cell responses to random checkerboard have been used to estimate their receptive fields and to find their location. The receptive fields of these cells tiled regularly the visual field (mosaic in Fig 1B).

To estimate noise correlation between pairs of cells, we computed the cross-correlation of their spike counts and the cross-correlation of their firing rates (the mean over stimulus repetitions of the spike count, respectively green and black lines in Fig 1C inset). Consistently with previous findings [30, 32], at short time-scales, spike-count correlation is larger than that of firing rates, but only for nearby pair of cells (Fig 1D). We term noise-correlation the difference between the zero-lag cross-correlation of spike counts and firing rate (Methods). We observed a similar behavior for all visual stimulations and experiments.

In the following we used these data to test our method. We first used copulas to predict the noise correlations between pairs of cells. We then used maximum entropy modelling to reconstruct the activity of a large population of ganglion cells from sequential recordings.

### Copula model predicts pairwise response from sequential recordings

**Fig 2:**
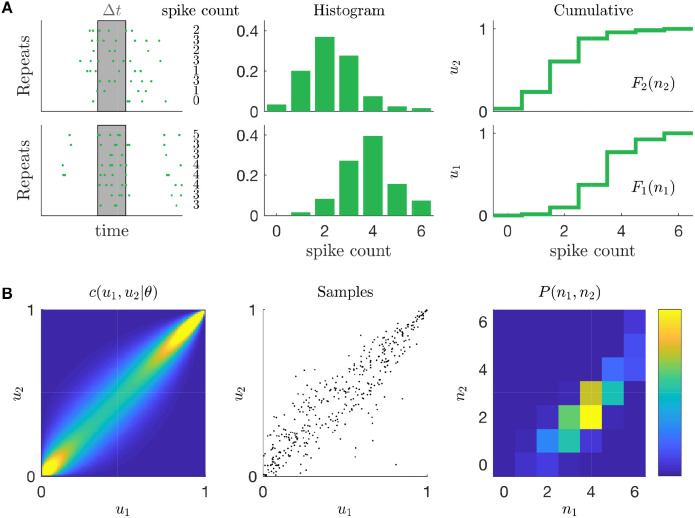
Schematic of the copula model. **A**) First step. Spike counts are estimated across stimulus repetition for a given pair of cells, in a given time-bin. Empirical histogram of the spike counts are then estimated and later used to compute the empirical cumulative distribution function. **B**) Second step. A copula distribution then accounts for the mutual dependency of two random variables. From it we draw many samples of pairs of real numbers (*u*_1_, *u*_2_) ∈ [0;1]^2^ with uniform marginals. Inverse of the cumulative distribution functions transform these samples into pairs of positive integer numbers, whose distribution matches the empirical spike count marginals by construction and accounts for their mutual dependency.

A copula is a method that can build pairwise probability distributions from pairs of single-variable distributions (Fig 2 and Methods). We used this approach to build the joint spike count distributions of pairs of neurons, that, if marginalized, reproduce the empirical single neuron distributions. For each time-bin, for each recorded neuron, we first estimated the spike count distribution from the response to stimulus repetitions, and from this we obtained its cumulative distribution function (Fig 2A). Next a copula model allowed us to compute the joint spike count distribution for all neurons pairs and time-bins using sampling (Fig 2B). Note that in our framework, the copula model and its parameter is the same for all time-bins.

**Fig 3:**
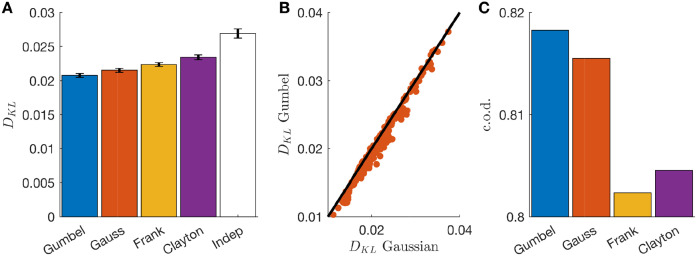
Gumbel copula outperforms other copula families. **A**) Kullback-Leibler divergence between empirical and model-predicted distribution for multiple copula families averaged over all cell pairs. **B**) Scatterplot of the Kullback-Leibler divergences for Gumbel and Gaussian families for each pair of cells. **C**) As **A** but using the c.o.d. for the prediction of noise correlations.

Pairwise parametric copulas are joint cumulative distribution functions (c.d.f.) characterised by model families with one or several parameters. Among the many possible copulas, a pairwise Gaussian copula is a joint c.d.f that takes a Gaussian form and is characterised by a correlation coefficient tuning the interaction between the two variables. It is possible to show (suppl. sect. S1 Text) that Gaussian copula modelling is equivalent to discrete dichotomized modelling over integer variables [46]. From this point of view, copulas with different model families can be seen as a generalisation of dichotomized Gaussians, with different functional forms for generating the correlation structure. To determine which was the best model family for our data, we tested several ones (independent, Gaussian, Gumbel, Frank and Clayton, see Methods) and estimate the Kullback-Leibler divergence with the empirical joint distribution. The Gumbel copula gave the best fits when tested on a stimulus (full-field) different from the one used for training (checkerboard) (Fig 3A) and the improvement over the second best, the Gaussian copula, was significant (t-test over the difference between the log-likelihoods, p-value ∼ 0, Fig 3B). Also the coefficient of determination (c.o.d) for the prediction of the correlation between each pair shows that the Gumbel copula has the larger performance (Fig 3C). Because of this result, we kept the Gumbel family for all the following analysis.

**Fig 4:**
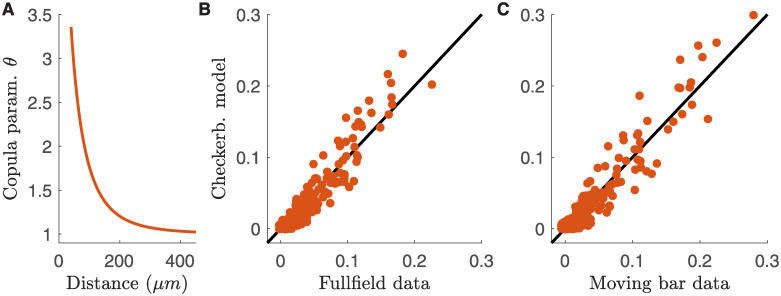
Copula model predicts noise correlations across stimulus ensembles. **A**) Gumbel copula parameter as a function of the inter-cell distance. **B**) Scatterplot of the empirical and model predicted noise correlations for the response to full-field stimulation. **C**) As **B** but for the moving-bar stimulation. In both cases the model has been inferred from the response to checkerboard stimulation.

A Gumbel copula is characterized by a parameter that tunes the interaction strength of the two variables, and that can be inferred from data. We found that this copula parameter only depended on the distance between the two cells, and can be fitted using a function with just three parameters (*θ* = exp(exp(*a* + *bx* + *cx*^2^)), where *x* is the distance between the two cells. Fig 4A, suppl. sect. S2 Text). We could thus describe the joint activity between all pair of neurons using copulas characterised by only three parameters across the entire population of cells. Once applied on the same response to checkerboard stimulation used for training, our model predicted noise correlations with high accuracy (Pearson’s *ρ* = 0.97, *n* = 300 pairs, larger than *ρ* = 0.92 obtained by fitting directly noise correlations (suppl. sect. S4 Text) and c.o.d. = 0.92).

We have built a model with only 3 parameters, that can predict the noise correlation between any pair of neurons from the activity of single cells. We then tested if this model can generalize and predict noise correlations measured in response to different stimuli. We first inferred the copula model from the response to checkerboard stimulation (that of Fig 1). Then, from the response to repetitions of another type of stimulus, we estimated the spike-count distributions of each neuron in each time-bin. Finally, we used our copula model to compute the mean noise-correlations of each neuron pair. We applied this strategy to the RGCs’ response to full-field and moving-bar stimuli (Fig 4B and 4C). In both cases, the copula model was able to reproduce the empirical estimates of noise-correlations with high accuracy (Pearson’s *ρ* = 0.95 and *ρ* = 0.95, c.o.d. = 0.84 and c.o.d. = 0.88 for full-field and moving-bar respectively, *n* = 300 pairs). In addition, in suppl. sect. S3 Text we show that our predictions are robust across a wide range of time-bins.

**Fig 5:**
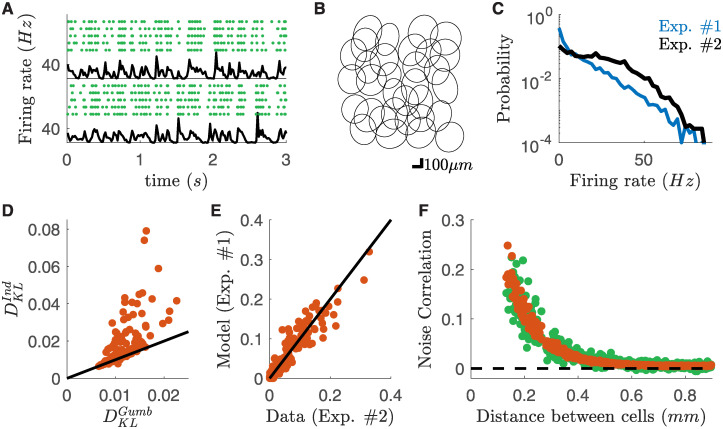
Copula model predicts noise correlations across experimental preparations. Data from a second dataset (#2), different from the one used for training the model (#1). **A**) Raster plots of two example cells in response to checkerboard stimulation for a second experiment. Each line corresponds to a repetition of the same visual stimulation. Black line: averaged firing rate of the cell. **B**) Receptive field mosaic of the recorded OFF cell population for the new dataset. **C**) Firing rate distributions over cells and time-bins for the two experiments. **D**) Scatterplot of the Kullback-Leibler divergence between empirical and model-predicted distribution for Gumbel and Independent copula models. Each point correspond to a different cell pair. **E**) Scatterplot of the empirical and model predicted noise correlations from the response to checkerboard stimulation. **F**) Behavior of the empirical and model predicted noise correlations plotted against the distance between cells.

To further demonstrate the robustness of our method we tested if our copula model could predict noise correlations in a different RGC population of the same type, recorded in a different experimental preparation (Fig 5A and 5B). Note that in the new experiments firing rates were higher than before (Fig 5C). We used the model inferred from the data of the first experiment to predict noise correlations between the same type of RGC, but recorded during a second experiment. Using only single cell responses, our model outperforms an independent copula model (Fig 5D) and its predictions were accurate (Fig 5E, Pearson’s *ρ* = 0.95 and c.o.d. = 0.91, *n* = 496 pairs), and accounted for how noise correlations decrease with distance (Fig 5F). The functional dependence of the copula parameter with respect to inter-cell distances is thus robust across experiments, and hence corresponds to a general property of OFF-Alpha cells in the rat retina. We obtained similar results for all the 8 testing experiments (overall Pearson’s *ρ* = 0.92 and c.o.d. = 0.80, for a total of *n* = 1633 pairs).

Note that in order to predict noise correlations (Fig 5) our model never accessed to the simultaneous recordings, but only to the collection of single neuron responses. We could have thus predicted the noise correlation between pairs of neurons in these new experiments using only the sequential recording of each neuron. Therefore, our approach allows for predicting pairwise noise correlations across experiments without requiring simultaneous recordings.

### Time-dependent maximum entropy model reconstructs the activity of large population from the copula’s pairwise predictions

Our copula model predicted pairwise synchronous firing. To reconstruct the activity of a large population of neurons from single cell recordings, we then used a *time-dependent* Maximum Entropy population model.

Standard Maximum Entropy models [31] aim at predicting the probability of any spike pattern from the mean firing rate of each neuron and the correlations between each pair of cells. Here we use a recent generalization of this approach [34, 35] that takes into account a time-varying firing rate. This approach builds a collection of pairwise Maximum Entropy models (one for each time-bin), which share the same couplings, but with different external inputs (fields) for each cell and each time bin [34, 35] (Methods). *Time-dependent* Maximum Entropy modelling thus disentangles intrinsic interaction, due to network effects, from extrinsic correlations, due to common inputs [35].

**Fig 6:**
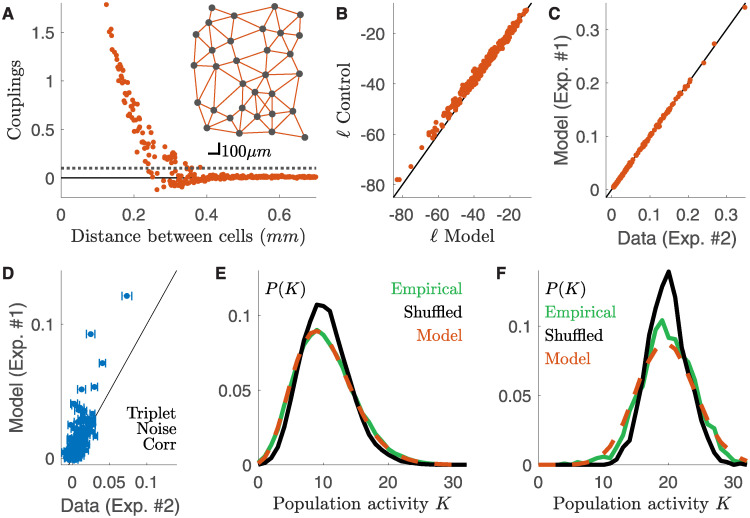
Time-dependent maximum entropy model predicts population synchronous firing across experimental preparations. Data from a second dataset (#2), different from the one used for training the model (#1). **A**) Behavior of the inferred couplings with the distance between cells. Grey dotted line threshold for identifying strong couplings. Inset: Position of the cells on the retinal surface, and strong couplings linking them. **B**) Scatterplot of the log-likelihoods of the copula-based model against those of a model inferred for data of the same experiment. **C**) Noise correlation prediction of the model against empirical value. As expected by construction, the model reproduces the copula estimations, and hence the empirical values (Fig 5B). **D**) Model prediction of triplet noise correlations against empirical value (*R*^2^ = 0.3, *n* = 4960). **E**) Empirical, shuffled (cond. independent) and model distributions of the population activity averaged over all time-bins. **F**) Same as **E** but for the 5% of time-bins with the highest mean population firing rate.

We inferred the *time-dependent* Maximum Entropy population model from the activity of single neurons and the pairwise correlations predicted by the copula model. The inferred couplings are large only between nearby cells, and the model reconstructs a “nearest-neighbour” interaction network (Fig 6A). We benchmarked the model against a time-dependent Maximum Entropy model inferred over the first half of the recording. Remarkably the model log-likelihood in each time-bin was only slightly smaller than the control (Fig 6B). As expected the model reproduced well the pairwise noise correlation (Fig 6C, Pearson’s *ρ* = 0.95, coefficient of determination cod = 0.91, *n* = 496 pairs), which were already finely predicted by the copula model (Fig 6E). Remarkably, Fig 6D shows that the model also partially accounts for the triplet noise correlation (Methods) (*R*^2^ = 0.3, *n* = 4960 triplets. *R*^2^ = 0.25 for the control time-dependent Maximum Entropy inferred on the first half of the data.). In order to show that our model captures the synchronous behavior of the neuronal population, we compute the probability distribution of the population rate, i.e. the total number of spikes emitted by the entire population in a given time-bin. We compared this distribution with the “shuffled” distribution, which destroys noise correlations, and is equivalent to the prediction made by a conditionally-independent model (i.e. a model that would assume there are no noise correlations). The distribution computed after shuffling the data overestimated the probability of number of spikes close to the average population rate, and underestimated the occurrence of transients of very large or very low activity (Fig 6E and 6F). Remarkably, our model captured the empirical behavior of the population rate averaged over the whole recording (Fig 6E) or when focusing only on highly active time-bins (Fig 6F).

### Synchrony behavior in a large population of ganglion cells reconstructed from multiple experiments

Thanks to our model, we could reconstruct the activity of a large population of neurons using only single cell activity. Since the model can generalize across experiments, it means that the activity of the different single cells can be taken from different experiments. Our method only needs a few pairs of neurons recorded simultaneously to fit the three parameters of the model. In the following we illustrate how this model can be used to reconstruct the activity from a very large population of neurons, bigger than what could be recorded experimentally. We illustrate how this inference of the activity of a large population of cells can be useful by measuring synchronous activity over increasing number of cells.

**Fig 7:**
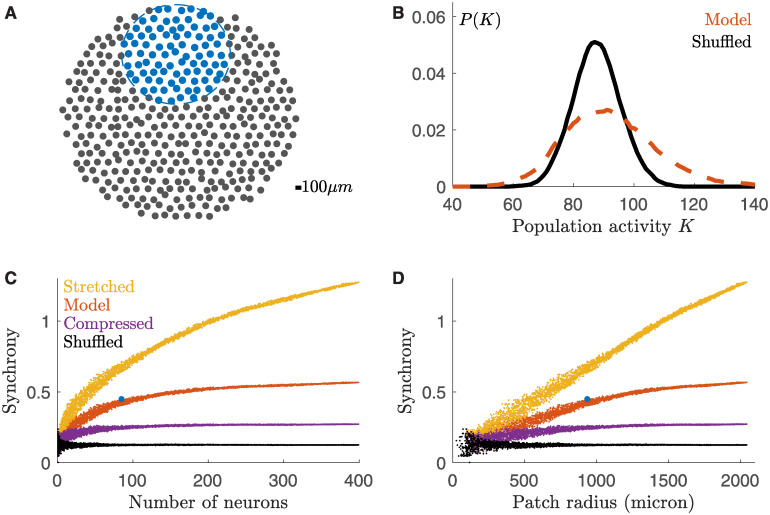
Large population model. **A**) Synthetic mosaic of *n* = 400 cells. Blue: example of sub-population. **B**) Probability distribution of the population activity for the time-bin with the highest firing rate. **C**) Population activity variance (synchrony) for many sub-populations against their number of neurons. Pairwise correlations are inferred either with our copula model (red), either with a stretched (yellow) or compressed (violet) model where the neurons correlate over a longer or smaller spatial scale. Black: cond. independent model without noise correlations. Blue point corresponds to the subpopulation of panel A. **D**) As **C**, but plotted with respect to the radius of the patch.

We first built a synthetic lattice representing the positions of the cells reproducing the empirical statistics of inter-cells distances (Fig 7A and Methods). Then, we collected the (marginal) response of many cells recorded during multiple experiments, and constructed a large population of *n* = 400 cells. We associated to each lattice position the response to ∼2*sec* checkerboard stimulation (repeated 79 times) of a randomly chosen cell among the ones recorded in all experiments (excluding those from the experiment used to learn the model parameters). Finally we applied our two-step approach to predict how this large population of cell would have responded to a checkerboard stimulus.

At first, we looked at the population rate as previously defined. In comparison with shuffled data, our model predicted a more frequent occurrence of transients with either very high or very low population activity (Fig 7B). This is a signature that synchronous activity extends up to large populations.

Then, to study how correlated firing grows with the population size, we sub-sampled the synthetic model and estimated the population synchrony. Note that in order to sub-sample the population, we did not choose neurons at random positions, but instead we randomly select a circle (by choosing a radius and a center location) and picked all the neurons within it (blue points in the example of Fig 7A). When enlarging the sub-population, new neurons are added at the border of the patch, and therefore can be very far from most of the others. Because in the data and in our model neurons are correlated only over finite distance (Figs 1D and 5F), the impact of a newly added cell on the population synchrony will strongly depend on the size of the sub-population. In case of a small sub-population, the added neurons will correlate with most of the present cells, thus having a large impact on the population synchrony. In case of a large sub-population instead, the added neurons will correlate with only with the nearest cells in the sub-population, and the population synchrony will not increase by much. We therefore expected a saturation of the population synchrony. We defined the population synchrony as the average over time of the variance of the population rate, divided by the population size (similar to the magnetic susceptibility in physics):

$$\begin{array}{c} \text{Synchrony}\equiv \frac{1}{N}{\left\langle \;\text{Var}\left(\sum_{i}n_{i}^{\left(t\right)}\right)\;\right\rangle }_{\text{time}}=\frac{1}{N}\sum_{ij}\;{\text{Cov}}_{\text{noise}}\left(n_{i}^{\left(t\right)},n_{j}^{\left(t\right)}\right) \end{array}$$ (1)

where $n_{i}^{\left(t\right)}$ is the spike count of neuron *i* at time *t*. As Eq 1 shows (see Methods for a derivation), the population synchrony can be estimated as the sum of all pairwise noise-covariances. For a large population, and for a fixed neuron *i*, only the terms corresponding to the nearest neurons will contribute to the summation in Eq (1). As a consequence, when increasing the population size, most of the new pairwise terms will not contribute to the sum, and the synchrony saturates. This saturation size depends mostly on the correlation length of the system. Red points in Fig 7C and 7D show that for our system this saturation happens at around 200 neurons, corresponding to a patch of radius 1.5*mm*, larger than what is experimentally accessible for synchronous recordings. To show how this saturation depends on the system correlation length, we repeated the same analysis after stretching (yellow points) or compressing (violet points) the copula parameter fit by a factor of two. The curve saturated later or before, respectively. When there is no noise correlation (shuffled data, black points), synchrony does not increase at all.

## Discussion

We have shown that our new method allows for reconstructing the population activity of large populations of neurons of the same type in the retina, based on sequential recordings and a few pairwise recordings. We have first developed a model to predict noise correlations across experiments. Thanks to this, once the model parameters are learned with paired recordings, we can take any pair of cells taken from new experiments, and predict the noise correlations from the activity of each single cell. Thanks to this prediction we could then reconstruct the activity of large populations of neurons of a single type at a scale beyond what can be recorded experimentally. Using this method, we have shown that synchrony in the population of rat OFF-alpha ganglion cells grows with the number of cells, becomes large and then saturates for very large populations. Understanding how the collective behaviour of neural ensembles scales with the number of neurons is a crucial issue, and our tool is a key method for this purpose, because it allows accurate inference of population activity, at a scale currently not accessible with experimental recordings.

Previous works have shown how different methods can be used to model and predict noise correlations. The Generalised Linear Model [15] uses spike history filters to couple the spiking activity between different neurons. Stimulus dependent maximum entropy models [34, 35] have coupling terms to model synchronous activity between pairs of neurons. These models have been successfully used to model how a population of neurons responds to a stimulus ensemble. However, they were never used to predict the activity of a population across different experiments. A main obstacle for this is that firing rates can vary from experiments to experiments, which would induce parameter changes in most of these models, and make generalization difficult. Here we have found that the noise correlation could be predicted using our model knowing just the distance between the two cells and their individual activity. Previous attempts did not allow using information on the marginal distribution of the spiking activity in the novel experiment. This flexibility is crucial in our method: our copula based model can include information of the marginal distributions straightforwardly, and this is one of its major advantages. Nevertheless, having a model that generalizes across experiments is crucial to pool together recordings from neurons of the same type and reconstruct activity of large neural ensembles.

On a broader perspective, our method can be included in the literature of attempts to predict missing correlations in a dataset. Woher and collegues [36], for example, propose to predict noise correlations as a function of stimulus correlations. Although this approach works for many stimulus ensembles, it will fail when, as in our elongated bar stimulations, stimulus correlations extend over large spacial scales, whereas noise correlations decrease sharply with distance (Fig 1). [37], instead, proposes to predict missing correlations when a large neural population is recorded through multiple measurements of overlapping patches. Yet, this method can not be applied easily to predict correlations in novel experiments. [38] reconstructs the trial-by-trial dynamics of large populations by extracting the common low-dimensional manifold. However this method also requires sequential but partially overlapping measurements, and thus suffers the same drawbacks. In a similar spirit, [39] assumes a common low-dimensional dynamics to predict the missing correlations in a large population recorded through multiple experiments. However, they find that some overlap between the recorded population is necessary for the stitching to work, and this exclude the application across different experiments. [40] combines data from different experimental sessions to infer the properties of the common underlying dynamical system, and then predict latent variables states across different experiments. However, even if it shows that the trial-to-trial variability follows the same principles across different populations, it’s not used to predict the variability in novel experimental settings.

Our approach to model noise correlations is based on copula distributions, and assumes that the copula parameter is constant across experiments. An alternative, simpler approach could have been to assume that noise correlations themselves remain similar across experiments. We constructed this simpler model by fitting an exponential function over noise correlations in our training dataset. This approach, however, gave significantly worse results than our method based on copulas (supplementary sect. S4 Text), demonstrating that our approach captures non-trivial properties of the correlated firing, i.e. its dependence on the firing rate of each cell. Previous works have shown that this dependence cannot be ignored and plays a major role in shaping the stimulus dependence of noise correlations [47]. Among the different proposed approaches, Dichotomized Gaussian, and their generalization to integer variables, have been shown to account with high precision for correlation patterns between spike counts [46]. In suppl. sect. S1 Text we have shown that Dichotomized Gaussians are mathematically equivalent to Gaussian copulas. The only difference is the method usually applied to infer the parameters (moment matching against maximum log-likelihood). For our data, Gumbel copula outperforms the Gaussian one (Fig 3), and therefore Dichotomized Gaussian. This result shows that with respect to previously proposed approaches, our copula based model provides an improvement in modeling noise correlations, and it is essential for the following steps of this work.

Remarkably, our description of noise correlation depends only on the physical distance between the pair. In suppl. sect. S2 Text, we have relaxed these assumptions and found that the copula parameter did not vary much with time, cell identity or stimulus. First, if we assume one copula parameter per time bin for a given pair of neurons, it varied little with time, and approached a constant value when the pairs’ firing rate were large enough. Second, when we inferred one parameter for each pair of cells to account for cell identities, their values still followed closely our parametric function. Finally, when we inferred copula parameters from the response to different stimulus ensembles, we obtained very similar values. This shows that the same copula distribution accounts well for the correlation between the two cells, independently of their firing rate, identities and of the stimulus ensemble.

Copulas have rarely been used in neuroscience studies [42–45, 48–50], but none of them applied this method to predict noise correlations. In [42] and [44], discrete copula distributions were used to model the total spike-count correlation in, respectively, pre-motor and pre-frontal cortex neurons. However, they did not distinguish stimulus from noise correlations as we have done here.

Our ability to generalize across stimuli and experiments—with similar mean luminance and contrast, but different statistics—demonstrates that the couplings between the recorded ganglion cells are insensitive to the context and depend mostly on the retinal adaptation state. This means that these couplings reflect an intrinsic property of the retinal circuit, and that the mechanism generating these noise correlations in not influenced by changes in the stimulus statistics or in overall firing rate. Since the timescale of the observed noise correlations is very fast (few ms, Fig 1C), the most likely mechanism is gap junctions, which create direct electrical connections between ganglion cells [51, 52]. Noise correlations might be more dependent on the context if they are generated by a shared noise source, e.g. the photoreceptor noise [53] or synaptic inputs [24]. Nevertheless, our results suggest that the strength of gap junctions are tuned to a value that seems preserved between different experiments—as long as mean luminance and contrast are kept constant [52].

An interesting outcome of our method is the possibility to construct models of arbitrary large populations, as long as enough sequential recordings are available. This possibility opens for testing a number of hypotheses on how correlated firing affects the overall population activity. Previous studies [31, 54] have made conjectures on the behavior of the retinal synchronous activity at large scale by extrapolating their results obtained upon sub-sampling the population of cells experimentally available. The validity of these extrapolations has been recently questioned [55], pointing out how the observed correlation pattern of small systems must be different from that of larger ones. In particular, [55] showed how sub-sampling a population may introduce spurious correlations that differ from those of a large population. Here we took the opposite strategy: in order to avoid the risk of sub-sampling, we built a large population model (Fig 7) pooling together real data and using our validated model to infer noise correlations between cells. Importantly, our method kept the spatial structure of correlations (spatial correlation scale), allowing for building realistic ganglion cells populations with the correct mosaic organisation. Our synthetic model thus provided the framework to further test the conjectures on the system’ behavior for large numbers of neurons, beyond what can be done experimentally.

We applied our method to the retina, where it is possible to have recordings of many neurons of the same type. In principle, our approach is general and could potentially be applied in other sensory area, provided that sequential recordings of the same type of cells are available, as well as some pairwise recordings to fit the model parameters. Further technological advances will make this method relevant to understand cortical populations, where it should be soon possible to define the cell type of each recorded cell using genetic (e.g. single cell transcriptomics [9, 10]) and physiological (e.g. clustering of responses [7]) tools. One issue could arise if noise correlations depend strongly on behavioural states, arousal or attention. In this case pooling together multiple recordings from different states may lead to heterogenous datasets that will not be well reproduced by our method. One step going in this direction has been made by [56], where correlations between cells appear correlated with the difference of their orientation preference, but only after conditioning on a state variable (in their case the global firing rate of the population). Computational approaches have also shown to be capable of accounting for these effects, for example by adding and learning an *external* modulator network that introduces a gain factor depending on running speed and pupil dilation [57]. Another issue could arise if noise correlations strongly depend on the stimulus, as it has, for example, been reported for V1 [58]. In our data, noise correlations depend very little on the stimulus, which allowed us to reduce our model and let copula parameters depend only on the distance between cells. However, if noise correlations depend largely on the stimulus, the model can be extended. The simplest solution would be to make the copula parameters depend on the stimulus. If this stimulus dependence can be explicitly modeled, our method would still manage to predict the activity of large ensembles of neurons. In addition, our method is based on pairwise copulas and pairwise Maximum Entropy models, and for analysing cortical activity, it might be necessary to account for higher order noise-correlations. In this case it will be necessary to extend our method with multivariate copulas [59] and higher order max-ent models [60].

Finally, here we predicted the responses of a population of neurons to a stimulus for which we have access to single cell responses across stimulus repetitions. If a model is available to predict the responses of single cell to other stimuli, it could be used to predict the marginal probability distribution of each individual neuron, and then combined with our approach to predict the activity of the whole population. This makes our method complementary to recent efforts trying to model and predict accurately the response of single neurons to complex stimuli in sensory areas [17, 57, 61].

## Methods

### Ethic statement

Animals were euthanized according to institutional guidelines in adherence with the Directive 2010/63/EU of the European Parliament, and approval was granted by the local ethical committee (CETEA n^*o*^5). Rats were sacrificed by CO2 inhalation followed by a quick cervical dislocation.

### Multi-electrode array recordings

We analyze the response of rat RGCs to visual stimulation recorded in 9 multi-electrode array *ex-vivo* experiments [3], from retina patches within 10-20 deg of eccentricity, and spike sorted with *SpyKING CIRCUS* [62]. This dataset and the experimental methods have been already previously described [18]. In one experiment we probed the retinal response to three different visual stimuli: (i) a random black and white checkerboard, with spatio-temporal uncorrelated checkers; (ii) a full-field stimulus with fluctuating luminance and (iii) two gray horizontal bars performing an independent Brownian motion along the vertical direction [18]. In the other 8 experimental sessions, only the response to random black and white checkerboard and full-field was retained and analyzed here. Each of these stimulations lasted about 10*sec* and have been repeated at least *R* = 79 times. Spiking times have been binned with a window of about 17*ms*, corresponding to a bin rate of 60*Hz*. With a custom algorithm [18] -similar to that of [7]- we used the cell’s response to the full-field stimulation to identify the type of the recorded RGCs. Across the 9 experiments, we identified populations of 20 ± 6 (mean ± s.d.) OFF-Alpha cells.

### Stimulus and noise correlations

After binning the spiking response of each cell, we estimate $n_{i}^{\left(t,r\right)}$, the number of emitted spikes by cell *i*, in the time-bin *t*, during repetition *r*, and its mean across repetitions $\mu_{i}^{\left(t\right)}$. Then we calculate the total covariance between two neurons (*i*, *j*) as follows:

$$\begin{array}{c} Cov_{total}\left(n_{i},n_{j}\right)=\frac{1}{T}\sum_{t=1}^{T}\frac{1}{R}\sum_{r=1}^{R}\left(n_{i}^{\left(t,r\right)}-\mu_{i}\right)\left(n_{j}^{\left(t,r\right)}-\mu_{j}\right) \end{array}$$ (2)

Where $\mu_{i}=\sum_{t=1}^{T}\mu_{i}^{\left(t\right)}/T$ is the mean number of spikes across repetitions, and then averaged in time. It is possible to decompose the total covariance into a sum of the so called “stimulus” and “noise” covariances. We calculated these quantities as follows

$$\begin{array}{c} Cov_{noise}\left(n_{i},n_{j}\right)=\frac{1}{T}\sum_{t=1}^{T}\frac{1}{R}\sum_{r=1}^{R}\left(n_{i}^{\left(t,r\right)}-\mu_{i}^{\left(t\right)}\right)\left(n_{j}^{\left(t,r\right)}-\mu_{j}^{\left(t\right)}\right) \end{array}$$ (3)

$$\begin{array}{c} Cov_{stimulus}\left(n_{i},n_{j}\right)=\frac{1}{T}\sum_{t=1}^{T}\frac{1}{R}\sum_{r=1}^{R}\left(\mu_{i}^{\left(t\right)}-\mu_{i}\right)\left(\mu_{j}^{\left(t\right)}-\mu_{j}\right) \end{array}$$ (4)

Noise correlations are then estimated as:

$$\begin{array}{c} Corr_{noise}\left(n_{i},n_{j}\right)=\frac{Cov_{noise}\left(n_{i},n_{j}\right)}{\sqrt{V_{i}V_{j}}} \end{array}$$ (5)

where *V*_*i*_ = *Cov*_*total*_(*n*_*i*_, *n*_*i*_).

Triplet noise correlations are instead defined as:

$$\begin{array}{c} Corr_{noise}\left(n_{i},n_{j},n_{k}\right)=\frac{\frac{1}{T}\sum_{t=1}^{T}\frac{1}{R}\sum_{r=1}^{R}\left(n_{i}^{\left(t\right)}-\mu_{i}^{\left(t\right)}\right)\left(n_{j}^{\left(t\right)}-\mu_{j}^{\left(t\right)}\right)\left(n_{k}^{\left(t\right)}-\mu_{k}^{\left(t\right)}\right)}{\sqrt{V_{i}V_{j}V_{k}}} \end{array}$$ (6)

### Copulas

Pairwise Copula-based modeling allows for disentangling the marginal distributions of two random variables from their mutual dependency, that can therefore be modeled alone, without the additional difficulties due to potentially complicated marginal distributions. Consider two random variables *X* and *Y*, with joint distribution *f*_*X*,*Y*_, marginal distributions *f*_*X*_ and *f*_*Y*_ and marginal cumulative distribution function (c.d.f.) *F*_*X*_ and *F*_*Y*_ respectively. By construction, the random variables *U*_*X*_ ≡ *F*_*X*_(*X*), *X* ∼ *f*_*X*_ and *U*_*Y*_ ≡ *F*_*Y*_(*Y*), *Y* ∼ *f*_*Y*_ have uniform distributions over [0, 1]. Consequently the joint distribution of (*U*_*X*_, *U*_*Y*_) has uniform marginals, yet it contains all the information about the mutual dependence between *X* and *Y*. This property allows us to model the dependency of *U*_*X*_ and *U*_*Y*_ *instead* of that of *X* and *Y*. Specifically, a copula is the c.d.f. of the joint variable (*U*_*X*_, *U*_*V*_), i.e. a function *C*(⋅, ⋅): [0, 1]^2^ → [0, 1]. and it can be used to reconstruct the joint distribution of (*X*, *Y*), via its c.d.f.:

$$\begin{array}{c} F_{X,Y}\left(x,y\right)=C(F_{X}\left(x\right),F_{Y}\left(y\right)). \end{array}$$ (7)

Sklar’s theorem (supplementary sect. S1b Text) ensures the existence and uniqueness of *C*, and this allows for modeling the mutual dependency between *X* and *Y*, independently from their marginal distributions. Note that when copulas are used to model discrete variables such as our spike counts, Sklar’s theorem still assures the existence of the copula, but its uniqueness is lost. However this is enough to model discrete joint distribution with copulas (suppl. sect. S1c Text).

### Parametric copula families

In this work we have considered the following parametric bivariate copula families:

- **Gumbel**:
  $$\begin{array}{c} C_{\text{Gumbel}}\left(u,v|\theta \right)=\text{exp}(\;-{\left(-{\left(\text{log}\;u\right)}^{\theta }+-{\left(\text{log}\;v\right)}^{\theta }\right)}^{1/\theta }\;), \end{array}$$ (8)
  where *θ* ∈ [1, ∞] is the copula parameter.
- **Gaussian**:
  $$\begin{array}{c} C_{\text{Gaussian}}\left(u,v|\theta \right)=\Phi_{\theta }(\Phi^{-1}\left(u\right),\Phi^{-1}\left(v\right)), \end{array}$$ (9)
  where *θ* ∈ [−1, 1] is the copula parameter, and Φ_*θ*_ is the joint cumulative distribution function of a multivariate normal with unit variance and covariance equal to *θ*. Φ is the cumulative of a standard normal distribution.
- **Frank**:
  $$\begin{array}{c} C_{\text{Frank}}\left(u,v|\theta \right)=-\theta^{-1}\;\text{log}\;(1+\frac{\left(e^{-\theta u}-1\right)\left(e^{-\theta v}-1\right)}{e^{-\theta }-1}), \end{array}$$ (10)
  where θ∈R is the copula parameter.
- **Clayton**:
  $$\begin{array}{c} C_{\text{Clayton}}\left(u,v|\theta \right)={\left[\text{max}\{u^{-\theta }+v^{-\theta }-1,0\}\right]}^{-1/\theta }, \end{array}$$ (11)
  where *θ* ∈ [−1, ∞]/{0} is the copula parameter.
- **Independent**:
  $$\begin{array}{c} C_{\text{Independent}}\left(u,v\right)=uv, \end{array}$$ (12)

We have inferred all the parameter values by log-likelihood maximization (suppl. sect. S2 Text). Once *θ* has been inferred, the marginal distributions can in turn be approximated either with some model, or, as we will do in this work, empirically. We refer to the Mathematics section, the literature and textbooks (see, for example, [41]) for more explanations and details on copula models and/or other copula families.

### Copula-based model

We constructed a copula model able to predict $P(n_{i}^{\left(t\right)},n_{j}^{\left(t\right)})$, the joint distribution of pair of spike counts in time:

$$\begin{array}{c} P({\left\{n_{i}^{\left(t\right)},n_{j}^{\left(t\right)}\right\}}_{t=1}^{T})=\prod_{t=1}^{T}c_{\text{Gumbel}}(F_{i}^{\left(t\right)}\left(n_{i}^{\left(t\right)}\right),F_{j}^{\left(t\right)}\left(n_{j}^{\left(t\right)}\right)\;|\;\hat{\theta }\left(d_{ij}\right)\;)\;, \end{array}$$ (13)

where *c*_Gumbel_ is the copula density function, corresponding to the c.d.f. of Eq (8), and $F_{i}^{\left(t\right)}\left(n_{i}^{\left(t\right)}\right)$ is the *empirical* c.d.f. of $n_{i}^{\left(t\right)}$, that we estimate across repetitions, *d*_*ij*_ is the distance between neurons *i* and *j*, and $\hat{\theta }\left(d_{ij}\right)$ is a parametric function: $\hat{\theta }\left(d\right)=\text{exp}\left(\;\text{exp}\left(a+bd+cd^{2}\right)\;\right)$ and $\hat{\theta }\left(d\right)=0$ if *d* > 1*mm*. To infer this function from data, we first inferred a copula parameter for each pair of neurons, *θ*_*ij*_, by log-likelihood maximization, and then we obtained *a* = 0.73, *b* = −0.014*μm*^−1^ and *c* = 810^−6^ *μm*^−2^ by fitting the behavior of *θ*_*ij*_ with respect to the distance *d*_*ij*_. See suppl. sect. S2 Text for further information.

### Time-dependent maximum entropy model

The time-dependent maximum entropy model we used is:

$$\begin{array}{c} P({\left\{\left\{n_{i}^{\left(t\right)}\right\}{)}_{i=1}^{N}\right\}}_{t=1}^{T})=\prod_{t=1}^{T}(\text{exp}\{\sum_{i}h_{i}^{\left(t\right)}n_{i}^{\left(t\right)}+\sum_{i\leq j}n_{i}^{\left(t\right)}J_{ij}n_{j}^{\left(t\right)}-\sum_{i}\text{ln}\left(n_{i}^{\left(t\right)}!\right)\}/Z^{\left(t\right)}) \end{array}$$ (14)

where $n_{i}^{\left(t\right)}\in \left[0,1,\ldots ,n^{\text{Max}}\right]$ is an integer spike-count, with *n*^Max^ matched from data. The index “^(*t*)^” expresses the time dependence. *Z*^(*t*)^ is a normalization constant (the partition function). $h_{i}^{\left(t\right)}$ is the local field for neuron *i* at time *t* imposing the firing probability and *J*_*ij*_ is the couplings network that allows for reproducing the system’s correlations. Note that *J*_*ij*_ does not depend on time and it includes also the diagonal terms *J*_*ii*_ which set each neurons variance equal to its empirical value [35, 63]. The log-factorial term allows for matching the single neurons statistics [63], as by taking *J* = 0 the model reduces to a collection of independent Poisson distributions.

### Inference of the time-dependent maximum entropy model

The inference of the model (14) is done by log-likelihood maximization using an iterative algorithm with adaptive learning rate similar to that of [64]. Because the model belongs to the exponential family, the log-likelihood derivative with respect to any parameter *θ*_*i*_ takes the form: 〈*O*_*i*_〉_data_ − 〈*O*_*i*_〉_model_, where *O*_*i*_ is the sufficient statistics conjugated to the parameter *θ*_*i*_. As a consequence, the model inference requires only the first two moments of $n_{i}^{\left(t\right)}$ across repetitions and the value of the noise covariances (Methods section c). In our case we estimate the firsts from the marginal response of each neurons and we used the copula model to predict the second, without any other empirical information or statistics.

### Synthetic lattice

In order to construct a synthetic lattice that respects the inter-cells distance of empirical recordings, we started by a triangular regular lattice with side 194*μm*, and then we added a Gaussian noise with a standard deviation of 22*μm* to the x and y coordinate of each cells. These parameters are optimized in order to match the distribution of cells distances measured in real experimental recordings.

### Synchrony as the population activity variance

We computed the population activity at time *t* as the sum over all the neurons of their spike-counts, $K^{\left(t\right)}\equiv \sum_{i}n_{i}^{\left(t\right)}$. We then defined the population synchrony as the average over time of the variance of *K*(*t*). It follows:

$$\begin{array}{ccc} \text{Synchrony} & \equiv & \frac{1}{N}{\left\langle \;\text{Var}\left(K^{\left(t\right)}\right)\;\right\rangle }_{\text{time}}=\frac{1}{N}{\left\langle \;\text{Var}\left(\sum_{i}n_{i}^{\left(t\right)}\right)\;\right\rangle }_{\text{time}}\\ & = & \frac{1}{N}\sum_{ij}{\left\langle \text{Cov}\left(n_{i}^{\left(t\right)},n_{j}^{\left(t\right)}\right)\right\rangle }_{\text{time}}=\frac{1}{N}\sum_{ij}\;{\text{Cov}}_{\text{noise}}\left(n_{i}^{\left(t\right)},n_{j}^{\left(t\right)}\right) \end{array}$$ (15)

## Supporting information

S1 Text Supplementary mathematics. (PDF)

S2 Text Supplementary information: Model construction. (PDF)

S3 Text Supplementary information: Time-bin dependence. (PDF)

S4 Text Supplementary information: Simplest model for noise correlations. (PDF)
