# Supplementary material for: Predicting synchronous firing of large neural populations from sequential recordings
Source: PLoS Comput Biol. 2021 Jan 28;17(1):e1008501. doi: 10.1371/journal.pcbi.1008501 (PMC7891787; doi:10.1371/journal.pcbi.1008501)
Supplement: S1 Text — (PDF) [file pcbi.1008501.s001.pdf]

S1. Supplementary mathematics

a Pairwise copulas. A pairwise copula function is the c.d.f. of a bi-variate distribution with $Uniform(0,1)$ marginals. This mathematical construct allows to model the dependency structure of bi-variate random variables separately from their marginal distributions, as the following theorem shows. Note that it is possible to extend copula for modelling also multivariate distributions, but here we focus only on the bi-variate case.

b Sklar's theorem. Let X and Y be any two, mutually dependent, real random variables. Let F_X, F_Y, and $F_{(X,Y)}$ be the c.d.f.s of X, Y, and (X,Y) respectively. Note that for any X we have $F_X(X) \equiv Uniform(0,1)$, *idem* for Y. The Sklar theorem asserts that given such X, and Y:

$$\exists! \text{ a copula } C, \text{such that } F_{X,Y}(x,y) = C(F_X(x), F_Y(y)) \tag{16}$$

c Discrete copulas. The proof of existence of Sklar's theorem holds for both continuous, and discrete random variables (as is our case). However, in practice, to apply copula models in the discrete case we need to make adjustments. In particular we turn a continuous copula, into a discrete distribution that may take values in a countable set of points in $[0,1]$. By doing so we define a so called "pseudo density" function, that describes a discrete counterpart to copulas. Assuming that $(X,Y) \in \mathcal{N}^2$, without loss of generality, the "pseudo density" of a copula defined by a copula density c is given by:

$$f_{pseudo}(F_X(x), F_Y(y)) = \int_{F_X(x-1)}^{F_X(x)} \int_{F_Y(y-1)}^{F_Y(y)} c(u,v)\, du\, dv \tag{17}$$

d Copulas and Discrete (Dichotomized) Gaussian. Discrete Gaussians (DG) are a generalization of Dichotomized Gaussians to account for integer-valued spike-count variables [46]. As it as been suggested ([46], sect. 3.3), here we show that Gaussian copulas are equivalent to DGs, and therefore copulas with arbitrary families can be seen as a generalization of DGs.

Let n_1 and n_2 be any two, mutually dependent, integer random variables. Let F_1, F_2, and $F_{(1,2)}$ be the c.d.f.s of n_1, n_2, and (n_1,n_2) respectively. Correlated samples of (n_1,n_2) - that reproduce their marginal - can be generated by first sampling from a Gaussian copulas density with correlation matrix Σ [41]:

$$(u_1,u_2) \sim c_\Sigma^{\text{Gauss}}(u_1,u_2) = \mathcal{N}[(0,0);(\Sigma^{-1} - \mathcal{I}_2)^{-1}]\left(\Phi^{-1}(u_1), \Phi^{-1}(u_2)\right) \ , \tag{18}$$

where $\mathcal{N}[\mu;\Sigma](x)$ is a normal (potentially multivariate) distribution of the random variable x with mean μ and covariance Σ, and Φ is the c.d.f of $\mathcal{N}[0,1](x)$. Then, samples of (n_1,n_2) can be obtained by $(n_1 = F_1^{-1}(u_1), n_2 = F_2^{-1}(u_2))$.

In the case of DGs, one first draws samples $(x_1,x_2) \sim \mathcal{N}[(0,0);\Sigma](x_1,x_2)$, and then transforms them as $(n_1 = \gamma_1(x_1), n_2 = \gamma_2(x_2))$, where $\gamma_i \equiv F_i^{-1} \circ \Phi$ [46], where $\circ$ states for function composition.

In order to prove the equivalence between Gaussian copulas and DGs, it is enough to split γ in two steps as $(v_1 = \Phi(x_1), v_2 = \Phi(x_2))$ and $(n_1 = F_1^{-1}(v_1), n_2 = F_2^{-1}(v_2))$, and observe

that (v_1, v_2) is distributed as (u_1, u_2) of Eq. (18), as can been seen by

$$P(v_1, v_2) = \int dx_1 dx_2 \, \mathcal{N}[(0,0);\Sigma](x_1, x_2) \, \delta\big(v_1 - \Phi(x_1)\big) \, \delta\big(v_2 - \Phi(x_2)\big) = c_{\Sigma}^{\mathrm{Gauss}}(v_1, v_2) \ , \quad (19)$$

where $\delta(x)$ is the Dirac delta function.
