# Supplementary material for: Predicting synchronous firing of large neural populations from sequential recordings
Source: PLoS Comput Biol. 2021 Jan 28;17(1):e1008501. doi: 10.1371/journal.pcbi.1008501 (PMC7891787; doi:10.1371/journal.pcbi.1008501)
Supplement: S2 Text — (PDF) [file pcbi.1008501.s002.pdf]

S2. Supplementary information: model construction

Here we fully justify how we simplified the copula model, from its bare version with one parameter for each neuron pair in each time-bin, to the final version with just three parameters in total.

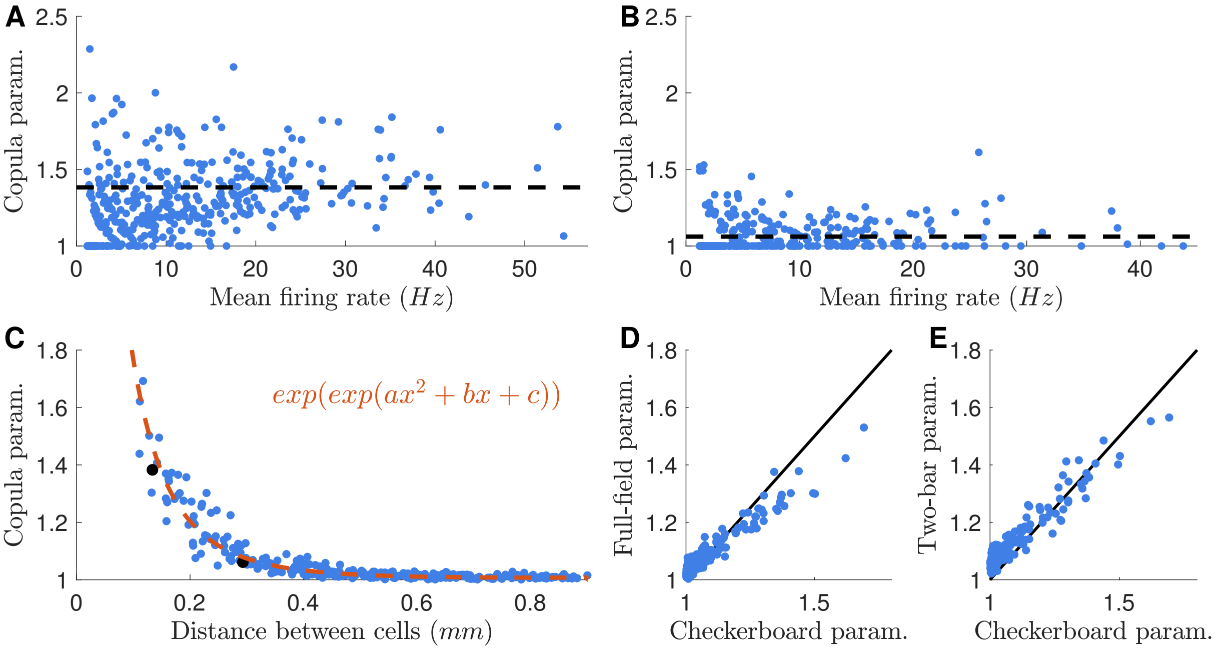

Stimulus-conditioned copula model is robust across time-bins and stimulus ensembles. A) Values of the inferred copula parameter in all time-bins for an example neuron pair, plotted against the mean firing rate of the two cells. Each point corresponds to a different time bin. Horizontal dashed line correspond to the inferred parameter in the *time-independent* copula model (see text) **B**) As **A**, but for another example pair. **C**) Inferred Gumbel copula's parameters plotted against the distance between cells for checkerboard stimulation. Parameters of each pairs are inferred independently. Orange: exponential fit used for estimating the copula parameter from the cell distance in the final version of the model. **D**) Scatterplot of the *time-independent* (see text) parameters inferred from checkerboard and full-field stimulations **E**) Scatterplot of the parameters inferred from checkerboard and two-bar stimulations

As explained in the Methods section, our starting point is a copula model where for each neuron pair (i, j) and each time-bin t a copula parameter $\theta_{ij}^{(t)}$ accounts for the correlation between the two spike-counts. Despite being very accurate, this model has little capacities to generalize across stimulus conditions or experiments. Moreover, because of the large number of parameters is potentially pruned to overfitting. In panels A and B, for two example neuron pairs, we show the values of the inferred parameters in all time-bins plotted against the mean firing rate of the two cells - in the corresponding time-bin and computed across repetitions. As can be observed, at high firing rate, that is when the statistics is large and the inference error is small, the inferred parameters tend to accumulate around a single value. This result suggested us that a model where the copula parameters does not depend on time could have

a similar performance, yet requiring much less parameters.

In order to infer such time-independent copula parameters, for each neuron pair, we first select the "active" time-bins where the two neurons spiked synchronously in at least one repetition: $\sum_{r=1}^{R} n_{i,r}^{t} n_{j,r}^{t} > 0$, where $n_{i,r}^{t}$ is the number of spikes emitted by neuron $i \in [1, \ldots, N]$, in time bin $t \in [1, \ldots, T]$ during repetition $r \in [1, \ldots, R]$. Once the inactive time bins are filtered out, we estimate the model parameter by maximizing the likelihood:

$$l(n|\theta) = \sum_{t} \sum_{r=1}^{R} \log(f_{pseudo}^{(t)}(n_{i,r}^{(t)}, n_{j,r}^{(t)}|\theta)) = \sum_{t} \sum_{r=1}^{R} \log \int_{F_i^{(t)}(n_{i,r}^{(t)}-1)}^{F_i^{(t)}(n_{i,r}^{(t)})} \int_{F_j^{(t)}(n_{j,r}^{(t)}-1)}^{F_j^{(t)}(n_{j,r}^{(t)})} c_{\theta}(u, v)\, du\, dv \tag{20}$$

where the summation over t runs over the active time-bins for the neuron pair. Note how F, and thus f_{pseudo}, depends on t, as the empirical marginals are estimated separately for every time bin. We infer the time-independent copula parameters θ_{ij} by log-likelihood maximization for each neurons' pair and for checkerboard, full-field and two-bar stimuli. Panels D and E compare the parameters inferred from different stimuli and show how the inference is robust across changes of visual stimulation. The copula parameter thus reflect some properties of the retinal network, independent of the current stimulus ensemble.

Panel C show the behavior of the inferred checkerboard parameters with respect to the physical distance between cells. Furthermore these parameters are independent of the visual stimulus (panels D and E). These results suggested us that a simple fit of copula parameters may account for most of the variability of the parameter values across neuron pairs.

To further simplify our copula model, and reduce the number of its parameters, we hence fitted the inferred copula parameters with a parametric function of the inter-cell distance dist: $\theta(\text{dist}) = exp(exp(a + b\,\text{dist} + c\,\text{dist}^2))$. The copula model takes now as input only the distance between the cells, uses it to estimate the copula parameter, and then construct the joint spike-count distribution using Eq. (13).
