# Supplementary material for: Predicting synchronous firing of large neural populations from sequential recordings
Source: PLoS Comput Biol. 2021 Jan 28;17(1):e1008501. doi: 10.1371/journal.pcbi.1008501 (PMC7891787; doi:10.1371/journal.pcbi.1008501)
Supplement: S3 Text — (PDF) [file pcbi.1008501.s003.pdf]

S3. Supplementary information: time-bin dependence

Here we study the performance of the Gumbel copula model depending on both the time-bin used for learning and testing. Specifically, we first learn the copula model from the responses to checkerboard stimulation binned at the "learning time-bin". Then we test it by predicting the noise correlation in responses to the full-field stimulation binned at the "testing time-bin". As it can be seen from the plot, the model performance is high and robust in the whole range 10-20ms. This result both validates our choice of 16.7ms (corresponding to 60Hz) and shows that the model is robust against changes of the working time-bin.

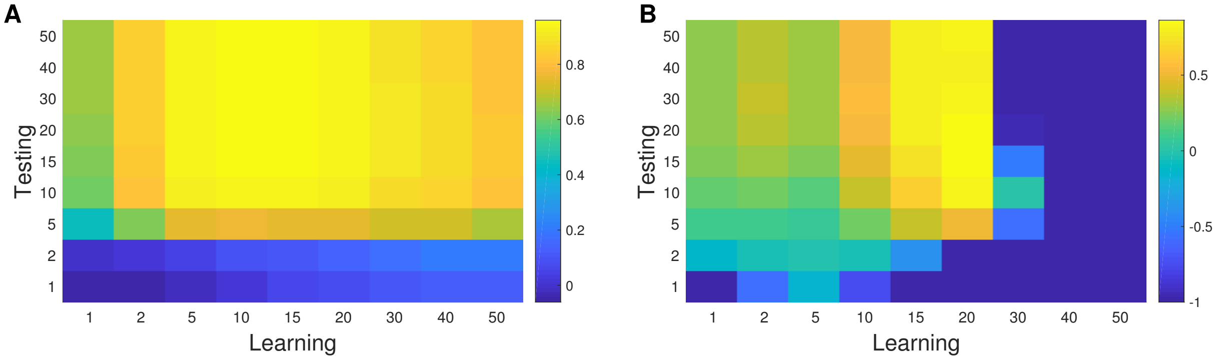

Performance of the model as a function of the time-bin used for learning the copula parameters on checkerboard stimulation and that used for testing on fullfield stimulation. **A**) Pearson correlation. **B**) Coefficient of determination (values smaller than -1 are set to -1 for readability purposes)
