# Supplementary material for: Predicting synchronous firing of large neural populations from sequential recordings
Source: PLoS Comput Biol. 2021 Jan 28;17(1):e1008501. doi: 10.1371/journal.pcbi.1008501 (PMC7891787; doi:10.1371/journal.pcbi.1008501)
Supplement: S4 Text — (PDF) [file pcbi.1008501.s004.pdf]

S4. Supplementary information: simplest model for noise correlations

In this section we compare the performance of our copula-based approach in predicting noise correlations with a straightforward model that assumes distant-dependent noise correlations.

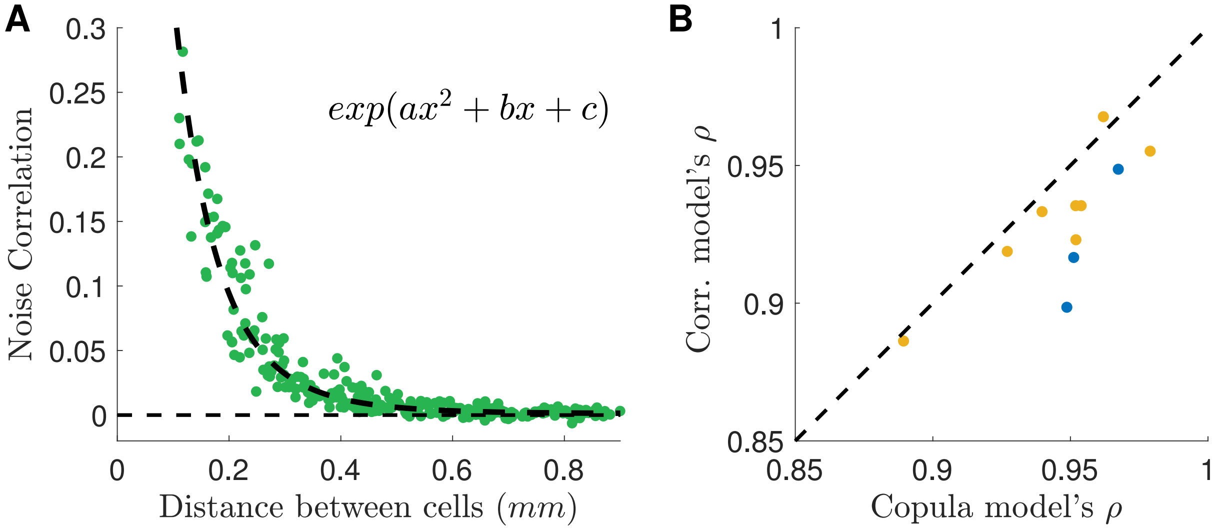

Copula model outperforms simpler model with distant dependent noise-correlations. A) Construction of the model: noise correlations observed in one experiment are fitted with an exponential function of the distance between neurons. Such fit is then used to predict noise correlations in other experiments. **B**) Performance in predicting noise correlation for our copula model against the model presented here. Blue points: performance for the first dataset with three different stimulus ensembles. Yellow points: performance for the other experimental sessions.

Noise correlations decrease with the distance between the corresponding neurons (see Fig. 1 and panel A above). We fit this relation with an exponential function, and we asked to what extent this behavior is conserved across experiments. To estimate this, we used this simple method to predict noise correlations in all the experiments described before. Although the predictions were accurate, our copula model outperforms this simpler approach (panel B).
